# Negative Bias During Early Attentional Engagement in Major Depressive Disorder as Examined Using a Two-Stage Model: High Sensitivity to Sad but Bluntness to Happy Cues

**DOI:** 10.3389/fnhum.2020.593010

**Published:** 2020-11-17

**Authors:** Xiang Ao, Licheng Mo, Zhaoguo Wei, Wenwen Yu, Fang Zhou, Dandan Zhang

**Affiliations:** ^1^Institute of Brain and Psychological Sciences, Sichuan Normal University, Chengdu, China; ^2^School of Psychology, Shenzhen University, Shenzhen, China; ^3^Department of Clinical Psychology, Shenzhen Kangning Hospital, Shenzhen, China; ^4^Shenzhen Institute of Neuroscience, Shenzhen, China; ^5^Shenzhen-Hong Kong Institute of Brain Science, Shenzhen, China

**Keywords:** major depressive disorder, attentional bias, mood congruent, dot-probe task 3, initial attentional allocation

## Abstract

Negative attentional bias has been well established in depression. However, there is very limited knowledge about whether this depression-relevant negative bias exits during initial attentional allocation, as compared with the converging evidence for the negative bias during sustained attention engagement. This study used both behavioral and electrophysiological measures to examine the initial attention engagement in depressed patients influenced by mood-congruent and mood-incongruent emotions. The dot-probe task was performed with a 100-ms exposure time of the emotional cues (emotional and neutral face pairs). The behavioral results showed that the patients responded faster following valid compared with invalid sad facial cues. Electrophysiological indexes in the frame of the two-stage model of attentional modulation by emotions provided cognitive mechanisms in distinct attention engagement stages: (1) the patients exhibited reduced P1 amplitudes following validly than invalidly happy cues than did the healthy controls, indicating a positive attenuation at an early stage of automatic attention orientation; and (2) the patients exhibited enhanced whereas the controls showed reduced P3 amplitudes following validly than invalidly sad cues, suggesting a mood-congruent negative potentiation in depression at the later stage of top-down voluntary control of attention. Depressed patients show a negative bias in early attentional allocation, reflected by preferred engagement with mood-congruent and diminished engagement with positive emotional cues/stimuli.

## Introduction

Major depression disorder (MDD) is a common mental disorder characterized by general and persistent depressed mood as well as loss of interest and enjoyment (American Psychiatric Association [APA], 1994). The MDD is a major contributor to the overall global burden of disease and is responsible for more “years lost” to disability worldwide than any other diseases (Smith and De Torres, 2014; Ferrari et al., 2016). According to the cognitive model of depression (Beck, 2008; Disner et al., 2011), a negative processing bias, reflected by extraordinarily enhanced reactivity to negative emotional cues or events (i.e., negative potentiation) and a reduced reactivity in response to positive emotional stimuli (i.e., positive attenuation), contributes to the onset, development, and maintenance of the disorder (Gotlib and Joormann, 2010). Typically, the negative attentional bias is a key component of the cognitive deficits; that is, MDD patients, when compared with healthy controls, demonstrate increased vigilance and selective attention to negative stimuli including negative facial expressions and words while they pay reduced attention to positive information (Mathews et al., 1996; Le et al., 2007; Leyman et al., 2007; Kellough et al., 2008; Fritzsche et al., 2010; Peckham et al., 2010; Yiend, 2010; Armstrong and Olatunji, 2012; García-Blanco et al., 2014; for reviews see Peckham et al., 2010; Yiend, 2010; Armstrong and Olatunji, 2012; Winer and Salem, 2016). It should be noted that the negative processing bias in MDD is distinct from the “negativity bias” (Ito et al., 1998) observed in healthy people. While the negativity bias developed during evolution denotes the enhanced emotional modulation effect of threat-related cues/stimuli (compared with positive and neutral stimuli) on one’s cognitive processes (Vuilleumier et al., 2001; Pessoa et al., 2002; Vuilleumier, 2005), the depression-relevant negative bias emphasizes on mood-congruent (i.e., sad) stimuli/events (Gotlib et al., 2004b; Koster et al., 2005; Arnone et al., 2012). For example, studies have revealed that MDD patients usually show attentional bias to sad facial pictures or depression-related words (Mathews and MacLeod, 2005; Peckham et al., 2010).

The dot-probe task (MacLeod et al., 1986; Brosch et al., 2008) with emotional stimuli as cues is frequently employed to investigate attentional modulation effects of emotion (MacLeod et al., 1986; Pourtois and Vuilleumier, 2006). In this task, pairs of stimuli (e.g., a fearful face and a neutral face) are briefly presented (as the cue) and immediately followed by a dot (as the probe) in the location of one of the previous stimuli. Participants are required to respond as quickly as possible to the probe. The rationale is that responses will be facilitated if the probe occurs in the location where participants already attend (Posner et al., 1980). The dot-probe task has been acknowledged as an effective tool to detect the impaired attentional bias in depressed patients (Peckham et al., 2010; Brown et al., 2014; Kappenman et al., 2014; Winer and Salem, 2016; Zhao et al., 2017).

Using this paradigm, previous studies have demonstrated that MDD patients often exhibit an attentional bias toward negative stimuli and/or away from positive stimuli when compared with healthy individuals (e.g., Gotlib et al., 2004a; Joormann and Gotlib, 2007; Zhou et al., 2015). However, it seems that the negative attentional bias in depression detected by the dot-probe task depends on the exposure time of the cues. While converging evidence has revealed that sustained negative attentional bias could be robustly observed with 1,000-ms or longer cue presentation durations (Mogg et al., 1995; Bradley et al., 1997; Gotlib et al., 2004a; Koster et al., 2005; Joormann and Gotlib, 2007; Fritzsche et al., 2010; Hankin et al., 2010; Günther et al., 2015; for review see Mathews and MacLeod, 2005; De Raedt and Koster, 2010; Gotlib and Joormann, 2010), there is no clear evidence of this negative attentional bias in MDD patients for short cue exposure durations (e.g., 100 ms), especially relevant for early attention engagement processes (Neshat-Doost et al., 2000; Donaldson et al., 2007). It has been proposed that the cue exposure time of 1,000 ms or longer allows more than one attention shift, and the negative bias found in this case indicates that depressed individuals show difficulties in disengaging attention from negative stimuli (Bradley et al., 1997; Gotlib et al., 2004a). To examine the negative bias reflecting an initial orienting toward negative stimuli, a 100-ms presentation time is suggested as the cue exposure time (Cooper and Langton, 2006). Considering the critical role of cue exposure durations on detecting attentional bias in depression, some studies have examined this factor using different exposure durations but obtained inconsistent conclusions. For example, Zhong et al. (2011) found that MDD patients exhibited a robust negative bias (increased attention to negative and decreased attention to positive stimuli) when the presentation time of cues was 500 ms, and there was also a trend of this bias when the presentation time was decreased to 100 ms. However, Trapp et al. (2018) reported that attentional bias toward painful facial expressions was only observed in depression at the presentation time of 100 ms rather than 500 ms, and the bias indexes at 100-ms condition were correlated with the depressive mood. Furthermore, some studies even observed the negative attentional bias using subliminally exposed cues (17 ms; Miskowiak et al., 2015). The null or non-significant finding at a short cue exposure time (e.g., 100 ms) in the aforementioned studies might be due to the affective materials used for the cues, that is, Donaldson et al. (2007) and Neshat-Doost et al. (2000) used negative words, and Zhong et al. (2011) used affective pictures selected from the International Affective Picture System (Lang et al., 2005). In our opinion, affective facial expressions are more suitable for detecting the negative attentional bias in the case of short cue exposure durations (Stevens et al., 2011; Syrjänen et al., 2019), because facial expressions contain inherent, salient, and evolutionarily adaptive information that should be rapidly perceived and responded by humans (Ekman, 1993).

Another possibility for the null finding at a short exposure time might be the relative insensitivity of the behavioral measure, e.g., the reaction time (RT). Furthermore, behavioral indexes alone could not reveal how the attention modulation of emotions works at the early perceptual stage and at the later top-down integration/regulation stage. The event-related potentials (ERPs) reflect brain responses directly with a high temporal resolution and is considered an ideal method for tracking the dynamics of neural activities in distinct cognitive stages. Using the ERP technique in the dot-probe task, our group has proposed the two-stage model of attentional modulation by emotions (Liu et al., 2015; Zhang et al., 2017): on the early stage (100–200 ms), the bottom-up pathway (involving the amygdala back-projects to sensory cortices) functions as a response scaling of sensory processing (reflected by the P1 component), which may magnify the sensory perception of emotional, compared with neural, information. On the later stage (250 ms and thereafter), the top-down integration pathway (involving the frontoparietal route to the amygdala) plays a critical role in modulating behaviors and regulating emotions (reflected by the P3 component). In light of these findings (Liu et al., 2015; Zhang et al., 2017), this study focused on the P1 and P3 components in depressed patients. In general, the occipital P1 (peaking at 100–150 ms post stimuli) is an attention-related component (Hillyard et al., 1998) and has been proved to be sensitive to early emotional modulation in visual perception (Schupp et al., 2007; Zhang et al., 2014). Meanwhile, the parietal P3 (peaking at 250–600 ms) is a task-relevant component (Polich, 2007) and often considered as an index of conscious and elaborate evaluation of emotional stimuli (Schupp et al., 2004).

The dot-probe studies focusing on healthy people have revealed that automatic attention attraction by emotionally stimuli reliably enhances the P1, while participants may also explicitly pay more attention to emotionally significant stimuli, reflected by an increased P3 (Pollak and Tolley-Schell, 2003; Pourtois et al., 2004; Brosch et al., 2008). As far as we know, three studies have combined the dot-probe task with ERPs to investigate the attentional bias in MDD patients. Among them, two studies (Hu et al., 2017; Li et al., 2018) selected a 500-ms cue exposure time and mainly examined the attention disengaging difficulty of patients (the P3), which found that the dot-evoked P3 amplitude was enhanced following sad faces in MDD participants. The other study (Zhong et al., 2011) used affective pictures and examined the P1 results in both 100- and 500-ms cue exposure conditions. The authors found that while the healthy group exhibited enhanced P1 amplitudes following positive pictures, this pattern did not appear in the MDD group; furthermore, the observed group difference was significant in the 500-ms condition and showed a trend in the 100-ms condition (Zhong et al., 2011).

As mentioned above, attentional biases consisting of increased attention to negative and reduced attention to positive stimuli are postulated in depression but have rarely been tested for early attentional processing (Trapp et al., 2018). In order to examine the negative bias during initial attentional orientation in MDD patients, this study set the cue exposure time as 100 ms and used mood-congruent (sad) as well as mood-incongruent (happy) facial expressions as the cue stimuli. We hypothesized that MDD patients would show depression-relevant negative bias during the initial attentional allocation stage besides during the well-known attention disengaging stage. In particular, patients would display enhanced responses to sad faces and may also show reduced processing of happy faces, which might be evidenced by an enhanced P1/P3 amplitude following sad faces and/or a reduced P1/P3 amplitude following happy faces when compared with healthy controls.

## Materials and Methods

### Participants

During the experimental design, we conducted *a priori* power analysis using G^∗^Power 3.1.7 (*F* tests, ANOVA: repeated measures, within-between interaction) based on the effect size (mean$\eta_{\text{p}}^{2}$0.12) reported in previous related studies (Zhong et al., 2011; Li et al., 2018). According to the result of this power analysis, 12 participants in total would ensure 80% statistical power. However, six participants per group is such a small sample size in present-day neuroscience studies. Thus, we finally decided to include 25 participants per group, which ensured a statistical power near 100%. As a result, 25 inpatients with MDD in Shenzhen Kangning Hospital and 25 healthy controls were recruited from clinics and through advertisements in the community. There was no significant difference between the two groups with respect to age, handedness, and education (Table 1).

**TABLE 1:** Demographic and clinical data of patient and control groups.

**Characteristics**	**Patient (*n* = 25)**	**Control (*n* = 25)**	**Statistics**
Mean age, years	37.3 (21–55)	38.0 (23–55)	*t*(48) = −0.26, *p* = 0.797
Education time, years	14.4 (9–19)	12.8 (9–19)	*t*(48) = 1.94, *p* = 0.059
Sex, male/female	12/13	13/12	
Handedness, right/left	25/0	25/0	
BDI-II	20.1 (14–48)	4.5 (0–9)	*t*(48) = 8.48, *p* < 0.001
STAI-S	41.9 (23–53)	37.4 (20–55)	*t*(48) = 1.70, *p* = 0.095
STAI-T	42.0 (28–65)	38.5 (20–55)	*t*(48) = 1.26, *p* = 0.215
Duration of illness, m	20.3 (0.5–180.0)		
Age at disease onset, years	32.5 (21–40)		
Number of lifetime episodes	2.0 (1–5)		

*Descriptive data are presented as mean (range). BDI-II, Beck Depression Inventory Second Edition (Beck et al., 1996); STAI-S/T, the State/Trait form of Spielberger’s State-Trait Anxiety Inventory (Spielberger et al., 1983).*

Patients were diagnosed with a current major depressive episode according to the *Diagnostic and Statistical Manual* (DSM-IV; American Psychiatric Association [APA], 1994). The diagnosis was based on structured clinical interview for DSM (SCID; First et al., 1996a) and chart review. In addition, all MDD participants were with a score of ≥14 on the Beck Depression Inventory Second Edition (BDI-II; Beck et al., 1996) in the time of experiment^1^. Exclusion criteria were neurological disorders and any comorbid Axis I disorders. Particularly, in view of the fact that anxiety and depressive symptoms are highly comorbid (Mineka et al., 1998; Kessler et al., 2005), and that many studies found a correlation between anxiety and abnormality of attention modulation of emotion (e.g., Mueller et al., 2009; Lin et al., 2015; Ho et al., 2017), we only recruited MDD patients without a diagnosis of anxiety disorder in this study. Furthermore, MDD patients with psychotic features, bipolar disorder, or Axis II disorders were excluded. The interview and clinical symptom rating were based on consensus of two senior psychiatrists who were trained with a relatively high reliability (κ = 0.87). At the time of experiment, the 25 patients were either untreated with any antidepressant medication or had undergone a washout period of at least 2 weeks.

Healthy control participants were screened for current Axis I and II disorders using the SCID-I/NP (First et al., 2002) and SCID-II (First et al., 1996b). They were additionally required to have a BDI-II score of ≤10. Exclusion criteria for both MDD and control participants were (1) seizure disorder, (2) history of head injury with possible neurological sequelae, and (3) substance abuse or dependence in the past 6 months. Participants were told about the content of the experiment. Written informed consent was obtained prior to the experiment. The experimental protocol was approved by the Ethics Committee of Shenzhen Kangning Hospital (code number: 2017-00264).

### Stimuli

Facial pictures were selected from the native Chinese Facial Affective Picture System (Gong et al., 2011), with an equal number of facial pictures of males and females. A total of 80 faces (20 happy, 20 sad, and 40 neutral faces) were used. Each picture had been assessed for its valence and arousal on a 9-point scale with a large sample of Chinese participants in a previous survey (Gong et al., 2011). The one-way ANOVA performed on the average scores of the 80 faces showed that the three categories of faces have significantly different emotional valence [*F*(2,77) = 143, *p* < 0.001, $\eta_{\text{p}}^{2}\,$ 0.787; happy = 5.92 ± 0.13; sad = 2.78 ± 0.13; neutral = 4.22 ± 0.09; pairwise comparisons: *p*s < 0.001] as well as arousal scores [*F*(2,77) = 30.2, *p* < 0.001, $\eta_{\text{p}}^{2}$ = 0.439; happy = 5.13 ± 0.22; sad = 5.83 ± 0.22; neutral = 3.82 ± 0.16; pairwise comparisons: emotional vs. neutral *p*s < 0.001, happy vs. sad *p* = 0.087].

All faces were gray-scale photographs. They were presented with the same contrast and brightness on the black background (3.0° × 3.5° visual angle). The target was a white dot (1.0° × 1.0° visual angle).

### Procedure

The experiment consisted of two blocks (happy and sad blocks), each containing 100 trials. The order of the blocks was counterbalanced across subjects.

**FIGURE 1:**
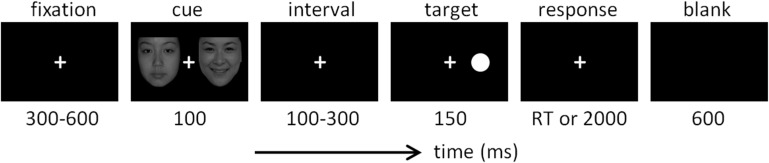
Illustration of one experimental trial in this study.

The design of the dot-probe task was very similar to that used in previous studies (Pourtois et al., 2004; Brosch et al., 2008; Liu et al., 2015). As shown in Figure 1, each trial started with a 300-to-600-ms fixation, followed by a 100-ms cue that consisted of two faces. In the happy block, the cue included a happy and a neutral face; in the sad blocks, the cue included a sad face and a neutral face. Each face was presented five times in a random order in corresponding blocks. The location of the neutral face in each trial was equal for the left or right side. After the cue and a short interval (100–300 ms), the target was presented with the duration of 150 ms. In valid trials, the target appeared at the location previously occupied by the emotional face; in invalid trials, the target appeared at the location previously occupied by the neutral face. Valid and invalid trials were presented in random order with equal probability (50 vs. 50%). Subjects were required to respond as quickly and as accurately as possible regarding the location of the dot target on the computer keyboard (the “F” key for the left location and the “J” key for the right location). The response screen would not disappear until a button press or until 2,000 ms elapsed. Responses with latencies of less than 2,000 ms were considered valid.

### Electroencephalography Recording and Analysis

Brain electrical activity was recorded referentially against left mastoid, by a 32-channel amplifier with a sampling frequency of 250 Hz (NeuroScan, Inc., Herndon, VA, United States). To make the results comparable with those of our previous study (Liu et al., 2015), the data were offline re-referenced to the average of the left and right mastoids. Besides electrooculogram electrodes, 30-channel electroencephalography (EEG) data were collected with electrode impedances kept below 5 kΩ. Ocular artifacts were removed from EEGs by using a regression procedure implemented in NeuroScan software (Scan 4.3).

The data analysis and result display in this study were performed by using Matlab R2011a (MathWorks, Natick, MA, United States). The recorded EEG data were filtered with a 0.1-to-20-Hz finite impulse response filter with a zero phase distortion. Filtered data were segmented beginning 100 ms prior to the onset of targets (dots) and lasting for 1,000 ms. All epochs were baseline-corrected with respect to the mean voltage over the 100 ms preceding the onset of targets, followed by averaging in association with experimental conditions.

We analyzed the amplitudes of occipital P1 and parietal P3 components across different sets of electrodes in accordance previous literature (Zhang et al., 2013, 2017; Luck, 2014; Liu et al., 2015; Gu et al., 2018). The mean amplitude of P1 was calculated at the electrode sites of O1 and O2 (time window = 120–150 ms). The mean amplitude of P3 was calculated at Pz, P3, P4, CP1, and CP2 (time window = 250–450 ms).

### Statistics

Statistical analysis was performed by using SPSS Statistics 20.0 (IBM, Somers, NY, United States). Descriptive data were presented as mean ± standard error. The significance level was set at 0.05. Three-way repeated-measures ANOVAs were performed on accuracy rate, RT, the P1 amplitude, and the P3 amplitude, with *emotion of the faces* (happy vs. sad) and *cue validity* (valid vs. invalid) as within-subject factors, and *group* (MDD patients vs. controls). Significant interactions were analyzed by using a simple-effects model. For the sake of brevity, the effects that did not reach significance have been omitted. Two-tailed Pearson’s *r* correlation was performed between depression score (measured by BDI-II) and behavioral/ERP data. Correction for multiple comparisons was based on Holm’s stepwise method.

## Results

### Behavioral Measures

#### Accuracy Rate

The accuracy rate (ACC) in all conditions was above 98%. The only significant effect was the main effect of cue validity [*F*(1,48) = 8.59, *p* = 0.005, $\eta_{\text{p}}^{2}$ = 0.152]: the accuracy was higher in the validly (99.2 ± 0.2%) relative to the invalidly cued condition (98.7 ± 0.3%).

#### Reaction Time

The main effect of *cue validity* was significant [*F*(1,48) = 20.6, *p* < 0.001, $\eta_{\text{p}}^{2}$ = 0.301; valid vs. invalid = 218.2 ± 7.8 vs. 223.9 ± 8.0 ms]. In addition, the interaction effect of *emotion* by *cue validity* was significant [*F*(1,48) = 5.11, *p* = 0.028, $\eta_{\text{p}}^{2}$ = 0.096].

**FIGURE 2:**
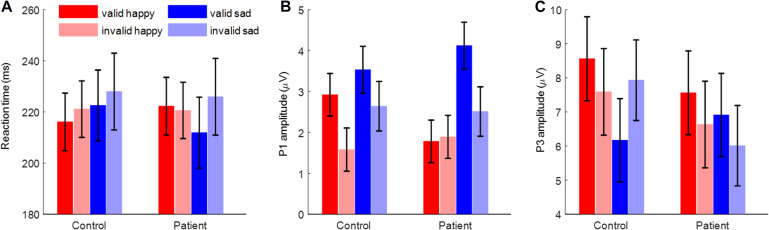
The reaction time, P1 amplitude, and P3 amplitude. **(A)** The reaction time. **(B)** The P1 amplitude. **(C)** The P3 amplitude. Bars represent standard error of the mean.

The most important finding is the significant interaction of *emotion* × *cue validity* × *group* [*F*(1,48) = 4.59, *p* = 0.037, $\eta_{\text{p}}^{2}$ = 0.087; Figure 2A]. To break down the three-way interaction, we tested the two-way interaction of *cue validity* × *group* in the happy and sad blocks separately. When the cue contained sad faces, not only the main effect of *cue validity* was significant [*F*(1,48) = 19.1, *p* < 0.001, $\eta_{\text{p}}^{2}$ = 0.285; valid vs. invalid = 217.2 ± 8.3 vs. 226.9 ± 9.1 ms], but also the *cue validity* × *group* interaction was marginally significant [*F*(1,48) = 3.73, *p* = 0.059, $\eta_{\text{p}}^{2}$ = 0.072]. Simple-effects analysis revealed that while patients responded faster for the validly than invalidly cued target [*F*(1,48) = 19.9, *p* < 0.001, $\eta_{\text{p}}^{2}$ = 0.293; valid vs. invalid = 211.9 ± 11.8 vs. 225.9 ± 12.9 ms], this effect was not found in the control group [*F*(1,48) = 2.98, *p* = 0.091, $\eta_{\text{p}}^{2}$ = 0.058; valid vs. invalid = 222.5 ± 11.8 vs. 228.0 ± 12.9 ms]. However, when the cue contained happy faces, neither the main effect of *cue validity* (*F* < 1) nor the two-way interaction was significant [*F*(1,48) = 2.45, *p* = 0.124, $\eta_{\text{p}}^{2}$ = 0.049].

### Event-Related Potential Measures

#### P1 Amplitudes

The main effect of *emotion* was significant [*F*(1,48) = 16.9, *p* < 0.001, $\eta_{\text{p}}^{2}$ = 0.260; happy vs. sad = 2.04 ± 0.32 vs. 3.20 ± 0.39 μV]. Also, the main effect of *cue validity* was significant [*F*(1,48) = 13.9, *p* = 0.001, $\eta_{\text{p}}^{2}$ = 0.224; valid vs. invalid = 3.09 ± 0.35 vs. 2.16 ± 0.35 μV].

**FIGURE 3:**
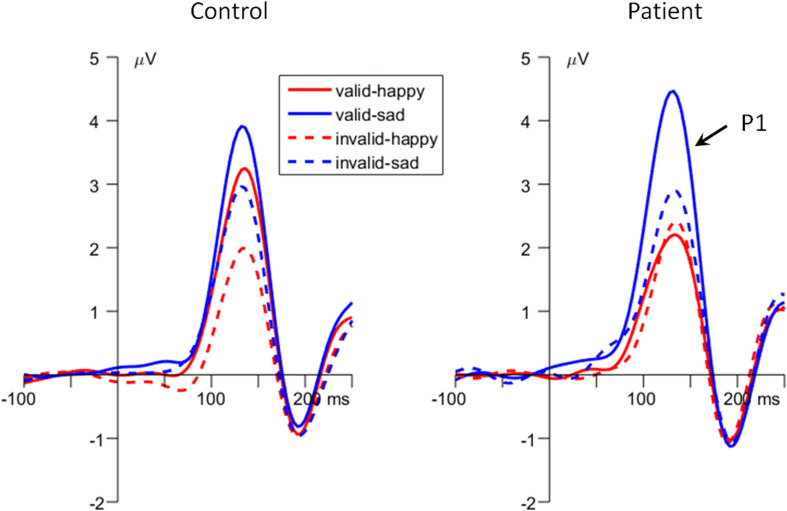
The grand-mean event-related potential (ERP) waveforms of P1 component in the two groups (data averaged at the occipital electrode site of O1 and O2).

Importantly, the interaction of *emotion* × *cue validity* × *group* was significant [*F*(1,48) = 5.27, *p* = 0.026, $\eta_{\text{p}}^{2}$ = 0.099; Figures 2B, 3]. To break down the three-way interaction, we tested the two-way interaction of *cue validity* × *group* in the happy and sad blocks separately. When the cue contained happy faces, the *cue validity* × *group* interaction was marginally significant [*F*(1,48) = 3.82, *p* = 0.057, $\eta_{\text{p}}^{2}$ = 0.074]. Simple-effects analysis revealed that while controls showed larger P1 amplitudes for valid than invalid happy cues [*F*(1,48) = 6.55, *p* = 0.014, $\eta_{\text{p}}^{2}$ = 0.120; valid vs. invalid = 2.92 ± 0.52 vs. 1.58 ± 0.53 μV], this effect was not significant in the patient group [*F*(1,48) < 1; valid vs. invalid = 1.78 ± 0.52 vs. 1.89 ± 0.53 μV]. However, when the cue contained sad faces, only the main effect of *cue validity* was significant for both groups [*F*(1,48) = 15.7, *p* < 0.001, $\eta_{\text{p}}^{2}$ = 0.246; valid vs. invalid = 3.82 ± 0.41 vs. 2.58 ± 0.43 μV].

#### P3 Amplitudes

The interaction effect of *cue validity* by *group* was significant [*F*(1,48) = 7.35, *p* = 0.009, $\eta_{\text{p}}^{2}$ = 0.133]. Also, the interaction effect of *emotion* × *cue validity* was significant [*F*(1,48) = 9.53, *p* = 0.003, $\eta_{\text{p}}^{2}$ = 0.166].

**FIGURE 4:**
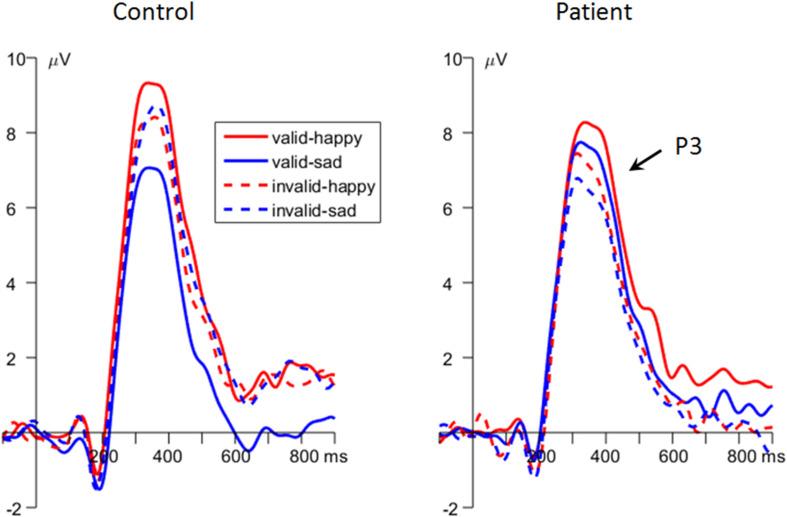
The grand-mean event-related potential (ERP) waveforms of P3 component in the two groups (data averaged at the parietal electrode site of Pz, P3, P4, CP1, and CP2).

Importantly, the interaction effect of *emotion* × *cue validity* × *group* was significant [*F*(1,48) = 9.25, *p* = 0.004, $\eta_{\text{p}}^{2}$ = 0.162; Figures 2C, 4]. To break down the three-way interaction, we tested the two-way interaction of *cue validity* × *group* in the happy and sad blocks separately. When the cue contained sad faces, the *cue validity* × *group* interaction was significant [*F*(1,48) = 24.9, *p* < 0.001, $\eta_{\text{p}}^{2}$ = 0.341]. Simple-effects analysis revealed that while patients showed larger P3 amplitudes for valid than invalid sad cues [*F*(1,48) = 5.73, *p* = 0.021, $\eta_{\text{p}}^{2}$ = 0.107; valid vs. invalid = 6.91 ± 1.23 vs. 6.01 ± 1.18 μV], the pattern was reversed in the control group; i.e., they had larger P3 amplitudes for invalid than valid sad cues [*F*(1,48) = 21.7, *p* < 0.001, $\eta_{\text{p}}^{2}$ = 0.311; valid vs. invalid = 6.17 ± 1.23 vs. 7.93 ± 1.18 μV]. However, when the cue contained happy faces, only the main effect of *cue validity* was significant [*F*(1,48) = 6.23, *p* = 0.016, $\eta_{\text{p}}^{2}$ = 0.115; valid vs. invalid = 8.06 ± 0.87 vs. 7.11 ± 0.90 μV].

#### Correlation

According to the results reported above, three correlations were performed between the depression score (measured by BDI-II) and the three behavioral/ERP indexes (i.e., the RT and the P3 amplitude in the sad condition, and the P1 amplitude in the happy condition). To directly examine the attention modulation of emotions, we calculated the difference measurement between validly and invalidly cued conditions (attentional bias score; see also Lubman et al., 2000; Townshend and Duka, 2001; Liu et al., 2015; Zhang et al., 2017).

**FIGURE 5:**
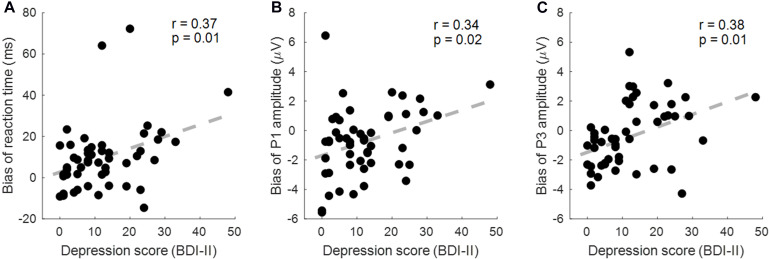
The correlation between the depression score [Beck Depression Inventory Second Edition (BDI-II)] and behavioral/event-related potential (ERP) indexes. **(A)** The reaction time (RT) bias (invalid–valid) in the sad cue condition is correlated with the BDI score. **(B)** The P1 amplitude bias (valid–invalid) in the sad cue condition was correlated with the BDI score. **(C)** The P3 amplitude bias (valid–invalid) in the sad cue condition was correlated with the BDI score.

As a result, the RT bias (invalid–valid) in the sad cue condition was correlated with the BDI score (*r* = 0.366, *p* = 0.009, corrected *p* = 0.018; Figure 5A). The P1 amplitude bias (invalid–valid) in the happy cue condition was correlated with the BDI score (*r* = 0.342, *p* = 0.015, corrected *p* = 0.015; Figure 5B). The P3 amplitude bias (valid–invalid) in the sad cue condition was correlated with the BDI score (*r* = 0.383, *p* = 0.006, corrected *p* = 0.018; Figure 5C).

## Discussion

While the negative attentional bias has been well established during attention maintenance and disengaging in MDD patients (for reviews, see Mathews and MacLeod, 2005; Peckham et al., 2010), there is no converging evidence for whether the negative bias exits during initial attentional orientation and what the cognitive mechanism is underlying this early attention process. Using a 100-ms cue exposure time in the dot-probe task, this study found that the MDD participants (but not the control ones) responded faster following valid compared with invalid sad facial cues, providing the second evidence (following Trapp et al., 2018) that depressed patients show a mood-congruent attentional bias during initial attention allocation. Our finding is consistent with the results reported by Rinck and Becker (2005), who used a visual search task and found that depression-related words were more distracting for the depressed than for control participants. In addition, we also found the main effect of cue validity for both behavioral measures; i.e., the validly cued condition was associated with a higher accuracy rate and a shorter RT than was the invalid condition, indicating a successful manipulation of cue validity in this study.

Beyond Trapp et al. (2018), this study contributes to the growing body of research aimed to reveal the underpinning of attentional bias in depression using ERP indexes that are sensitive to distinct attention allocation stages. Our results show that the MDD patients exhibited enhanced P1 amplitudes following validly than invalidly sad cues, whereas the healthy controls showed enhanced P1 amplitudes for both validly (compared with invalidly) happy and sad cued targets. The P1 has been associated with rapid and unconscious bottom-up attention allocation (Luck and Kappenman, 2012). Previous ERP studies have demonstrated that automatic attention attraction by emotionally significant stimuli could reliably enhance the P1 amplitudes in the dot-probe task, representing a facilitated perception in response to the target presented at the already attended location (Hopfinger and Mangun, 1998; Pourtois et al., 2004; Brosch et al., 2008; Liu et al., 2015). The P1 finding of this study suggests that during an early stage of bottom-up response scaling of sensory processing, MDD patients show decreased attention for positive stimuli than do healthy people. This result is in line with the evenhanded theory (Gotlib and McCabe, 1992; McCabe and Gotlib, 1995), which argued that while healthy people are usually more desirable to attend to positive stimuli (compared with neutral ones) and display optimistic bias that is self-protective and good for mental health, depressed people, by contrast, lack this protective, positive bias, so they show no difference in attentional allocation between positive and neutral information (Korn et al., 2014; Winer and Salem, 2016). The current P1 results not only are consistent with previous behavioral findings in depression (Gotlib et al., 1988; Mogg et al., 1991; Koster et al., 2005; Kellough et al., 2008; Atchley et al., 2012; for reviews see Peckham et al., 2010; Winer and Salem, 2016) but also restrict the attentional impairment within the P1 time window, i.e., on the stage of automatic bottom-up attention allocation.

Unlike the P1, the P3 component has been proven to reflect top-down modulation and voluntary allocation of attention (Fichtenholtz et al., 2007; Polich, 2007; Leutgeb et al., 2015), and a larger P3 in the dot-probe task indicates explicit attention being paid to emotionally significant stimuli (Pollak and Tolley-Schell, 2003). The main finding at the P3 of this study is that the MDD patients exhibited enhanced P3 amplitudes following validly than invalidly sad cues, whereas the control participants showed an opposite results, i.e., displayed enhanced P3 amplitudes following invalidly than validly sad cues. According to the two-stage model of attentional modulation by negative emotions (Liu et al., 2015; Zhang et al., 2017), the attention modulation of emotions at the later stage is voluntarily controlled; thus, attention could be flexibly tuned to fit cognitive goals with the least effort. Therefore, the enhanced P3 for invalid sad cues in the control group indicated that more cognitive resources were allocated toward the task-relevant but previously less attended location, in order to ensure the effective achievement of task goals. However, the MDD patients paid more attention to valid than invalid sad cues on both stages of automatic and voluntary attention allocation, indicating a negative, mood-congruent attention bias in depression (Peckham et al., 2010; Yiend, 2010; Armstrong and Olatunji, 2012; Koster and Hoorelbeke, 2015; Winer and Salem, 2016). Similar with this finding, other studies using relevant tasks also reported enhanced attention and P3 amplitudes for negative stimuli in depressed as compared with healthy individuals (Atchley et al., 2003; Ilardi et al., 2007). In addition, this study found that on the stage of top-down voluntary modulation of attention, both MDD patients and healthy controls showed enhanced P3 amplitudes following validly than invalidly happy cues, though due to distinct reasons. While the patients reallocated more attention toward the previously less attended happy-cued target in order to perform well in the task, healthy participants continued to assign more attention resources to the location of happy compared with neutral cues due to their protective, positive bias (Gotlib and McCabe, 1992; Korn et al., 2014; Winer and Salem, 2016).

Taken together, the findings of behavioral and ERP measures indicate that MDD patients show mood-congruent bias in early attention allocation processes, reflected by the sad-prone indexes of RT and P3 amplitudes, and the happy-avoidance index of P1 amplitudes. Interestingly, the attentional bias scores of these three indexes were correlated well with the depression level measured by the BDI-II (Figure 5). These findings have valuable clinical implications for both impairment titration and timely evaluation of treatment effects. For instance, many studies used the procedure *attention bias modification* to alleviate the negative bias in depression (for reviews, see Hallion and Ruscio, 2011; Mogoaşe et al., 2014), which found that reducing the attentional bias score based on RT could contribute to the recovery process for MDD patients (Zhou et al., 2015) and causally reduce the chance of depressive recurrence (Browning et al., 2012). This study further provides electrophysiological indexes in complementary with previously employed behavioral ones. The advantage is that the attentional bias scores based on the amplitudes of the P1 and the P3 could separately and specifically tell whether and to what extent the deficits in automatic (the P1) and voluntary (the P3) attentional allocation are in patients, so as to provide a more detailed instruction for individualized treatment.

Besides clinical implications, the current findings also facilitate the understanding of cognitive mechanism of attentional modulation by emotions. Previously, we used threat-related facial expressions as cues (anger, fear, and disgust) and proposed the two-stage model of attentional modulation by emotions (Liu et al., 2015; Zhang et al., 2017). In this study, the examination of another two basic emotions, i.e., sadness and happiness, added important knowledge to this cognitive model. On the early stage of bottom-up response scaling of sensory processing (reflected by the P1), the brain magnifies (anger, fear, sadness, and happiness) or minifies (disgust) the sensory perception of emotions by automatically allocating increased or decreased attention resources toward the location of emotional stimuli, in accordance with the evolutionary purposes of various emotions. On the later stage of top-down attention control (reflected by the P3), attention is voluntarily tuned to fit task goals with least effort (negative emotions) or maintained upon the emotional stimuli in order to achieve a status of mental well-being (positive emotions).

Finally, one limitation of this study should be noticed. Although a large amount of studies have shown that a 1,000-ms cue could reliably evoke negative attentional bias in depression, it is preferred to directly compare the attentional bias effects in 100- and 1,000-ms conditions within one study. This could be one of the further directions to facilitate a comprehensive understanding of the negative attentional bias in depression.

## Conclusion

In conclusion, this study examined the depression-relevant negative bias during initial attentional allocation in MDD patients. The ERP measures revealed that (1) during an early stage of automatic attention allocation (reflected by the P1 amplitude), MDD patients showed decreased attention for positive stimuli than did healthy people; and (2) they allocated enhanced attention toward mood-congruent sad stimuli than did controls on the later stage of top-down voluntary control of attention (reflected by the P3 amplitude). The attentional bias scores of the P1 and the P3 amplitudes were correlated with the depression levels, providing electrophysiological indexes in distinct (early automatic vs. later voluntary) attentional allocation stages to guide the clinical treatment of depression. Furthermore, the findings in the healthy control group enriched the two-stage model of attentional modulation by emotions and deepened our understanding of cognitive mechanism underlying the emotional influence on attention.

## Data Availability Statement

The original contributions presented in the study are included in the article/supplementary material, further inquiries can be directed to the corresponding author.

## Ethics Statement

The studies involving human participants were reviewed and approved by the Ethics Committee of Shenzhen Kangning Hospital. The patients/participants provided their written informed consent to participate in this study.

## Author Contributions

DZ and ZW designed the study. ZW, WY, and FZ collected the experimental data. DZ analyzed the data. XA and LM wrote the first draft of the manuscript. All authors revised the manuscript.

## Conflict of Interest

The authors declare that the research was conducted in the absence of any commercial or financial relationships that could be construed as a potential conflict of interest.

## Footnotes

1 Note that while BDI-II < 14 indicates minimal depression, BDI-II ≥ 14 indicates mild, moderate, or severe depression.
